# Predicting median nerve depth from anthropometric features: A tool for safer invasive procedures

**DOI:** 10.1371/journal.pone.0330383

**Published:** 2025-08-18

**Authors:** Sara Mogedano-Cruz, Ángel González-de-la-Flor, Cristina Rodríguez-Anadón, Lucimere Bohn, Jorge Villafañe, Carlos Romero-Morales

**Affiliations:** 1 Department of Physiotherapy, Faculty of Medicine, Health and Sports, European University of Madrid, Villaviciosa de Odón, Madrid, Spain; 2 Lusófona University, Porto, Portugal; Fondazione Policlinico Universitario Gemelli IRCCS, ITALY

## Abstract

**Introduction:**

The median nerve (MN) is frequently targeted in invasive procedures. Accurately locating its depth is essential to minimize complications. This study aimed to develop predictive models of MN depth based on anthropometric features. Design: cross-sectional observational study.

**Methods:**

Fifty-three healthy adults (Men: 53%; Age range: 18–60 years) were evaluated. Sociodemographic (age and sex) and anthropometric data (height, weight, BMI, and proximal/mid-forearm circumference) were ascertained. Ultrasound was used to measure the depth of the MN relative to the skin and brachial artery at the elbow and mid-forearm. Hierarchical linear regression was applied to identify significant predictors of nerve depth.

**Results:**

Men were significantly taller, heavier, and had a higher forearm circumference than women (p < 0.05 for all). Proximal forearm circumference strongly correlated with BMI and nerve depth. Regression analysis revealed it as a significant predictor of MN depth, explaining 49.4% (proximal) and 95.2% (mid-forearm) of the variance. The model for nerve-to-artery distance showed limited explanatory power (R^2^ = 0.164).

**Conclusion:**

The mid-forearm circumference is a strong and accessible predictor of MN depth. The proposed models can support clinicians in estimating appropriate needle depth in ultrasound-guided procedures, potentially reducing the risk of nerve injury.

## Introduction

The median nerve (MN), derived from the medial and lateral cords of the brachial plexus, receives contributions from the C5-T1 nerve roots. It descends along the arm lateral to the brachial artery, passing between the biceps brachii and brachialis muscles. In the antecubital fossa, it traverses the bicipital aponeurosis and positions itself anterior to the brachialis muscle. It then passes between the superficial and deep heads of the pronator teres (PT) to settle in the forearm between the flexor digitorum superficialis (FDS) and flexor digitorum profundus (FDP) muscles [1].

Throughout its course, the MN may become compressed at various points, leading to different neuropathies. Common compression sites include the area beneath the Struthers’ ligament, the bicipital aponeurosis, several points between the heads of the pronator teres, and the fibrous arch of the FDS [1–3]. Electrophysiological studies and ultrasound are essential for identifying the injury site and assisting in appropriate medical or surgical management decision-making [4].

Regarding treating these nerve injuries, electrical nerve stimulation has been found to modulate the pain control system in the dorsal horn, helping to reduce neuropathic symptoms. Depending on the depth of stimulation, two types of application can be distinguished: transcutaneous electrical nerve stimulation (TENS) and percutaneous electrical nerve stimulation (PENS) [5,6]. In TENS, electrodes are placed on the skin, and electrical impulses are applied to stimulate nerve endings. In contrast, PENS involves placing electrodes in the subcutaneous space, enabling more precise and localized nerve stimulation under ultrasound guidance [5].

Recently, the use of PENS has increased significantly. Although these techniques are generally safe and effective, they can cause minor adverse effects, such as bruising and pain during or after the procedure. Additionally, in rare cases, more serious adverse effects may occur [7]. Therefore, the risk of nerve and vascular tissue damage remains a critical concern in clinical practice.

Ultrasound-guided identification and localization of the target tissue before performing PENS techniques can help prevent needle-induced injuries. Additionally, achieving a consistent response to electrical stimulation with needles can be challenging due to factors such as variations in electrode placement, skin movement, and physiological differences, all of which affect tissue conductance [5,8]. For this reason, it is essential to develop a clinical protocol that accounts for these variations and ensures reproducible outcomes.

Ultrasound is a non-invasive imaging diagnostic tool used to examine tissue structure [5,9]. The ultrasound probes consist of small transducers that convert electrical energy into sound pulses, which are then reflected back to the transducer and converted into electrical signals. A software analyzes these signals and generates a real-time two-dimensional image. Additionally, the echo intensity, which reflects the amplitude of the sound pulse, allows for tissue characterization as it depends on the difference in sound conduction speed in adjacent structures. This variation in intensity helps distinguish subtle details between different biological tissues [10].

The use of ultrasound to visualize the MN has become increasingly common due to its accessibility, low cost, non-invasiveness, and relatively short examination times [5,9,11]. While ultrasound does not provide information about nerve function, it is useful for observing variations in size, shape, echogenicity, and vascularity [11]. However, its use requires both access to equipment and specific training. In such contexts, being able to estimate nerve depth using simple, external anthropometric measures may improve both the precision and safety of invasive interventions.

The main objective of this study was to identify the cross-sectional area (CSA) and depth of the MN at two specific locations, as well as to establish a linear distance relationship with adjacent vascular structures, considering age, sex, weight, height, body mass index (BMI) and the circumference of the area to be assessed. The linear regression model could identify statistically significant predictors of these MN variables in healthy subjects to prevent potential damage caused by puncture during invasive procedures.

## Methods

### Study design

This cross-sectional observational trial was carried out with a convenience sample.

The study protocol followed the guidelines and checklist of Strengthening the Reporting of Observational Studies in Epidemiology (STROBE).

### Ethics statement

The protocol study was approved by the Universidad Europea de Madrid Committee (CI/2024–877), and all procedures followed the Declaration of Helsinki. All participants signed the informed consent form and received a patient information sheet (Supplementary data S1 File).

### Participants

Fifty-three participants were recruited between December 2024 and February 2025 from the Universidad Europea Labs. Participants should be aged between 18 and 60 years old. Exclusion criteria included any pathology or history of surgery in the upper limb, neuromuscular conditions affecting normal tissue morphology, pregnancy, and performance-enhancing drugs.

### Sample size calculation

A prior sample size was estimated following Beneziuk et al. [12] recommendation. According to them, 10–15 participants must be assessed for each potential predictor, and the number of predictors must be limited to 5 to avoid overestimating the precision [13]. Based on that, we obtained a sample size of 53 participants.

### Demographic and anthropometric data

Sociodemographic data (sex and age) were ascertained with interviews. Body height (cm), weight (kg), and body mass index (BMI; weight/height^2^)) were established according to protolocs published elsewhere [14].

Additionally, the right proximal and mid forearm circumferences were measured using a tape and recorded in cm. The proximal forearm was measured at theelbow and at the mid-forearm, at the midpoint between the elbow and the wrist.

### Ultrasound imaging procedure

The subjects were supine with the right elbow fully extended and the forearm in supination, resting on the examination table. All ultrasound exams were performed by a single examiner experienced in musculoskeletal ultrasound, using a Sakura P10 ultrasound system (SonoScape, Shenzhen, China) equipped with a 12L-B linear transducer. Ultrasound settings, including frequency, gain, focus, and dynamic range, were individually optimized to ensure the highest image quality.

**Fig 1:**
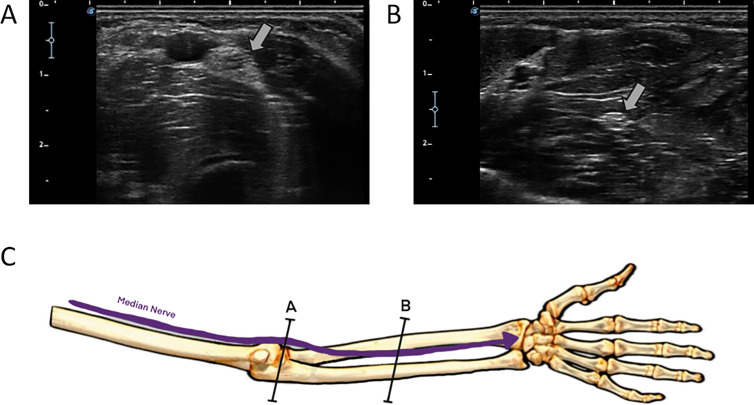
Ultrasound images of the MN at the proximal third (A) and middle third (B) of the forearm. The obtained images show the measurement sites used for morphometric analysis.

During the examination, minimal pressure was applied to the transducer to avoid any distortion or compression of the tissues. The transducer was kept perpendicular throughout the procedure to prevent anisotropy [4]. Three measurements were taken for each participant, with the average value used as the final measure. The ultrasound device’s zoom function was not used during the assessments to avoid potential bias in the measured values. Fig 1 shows the ultrasound images obtained at both the elbow and the mid-forearm.

The proximal ultrasound examination of the MN was performed in the medial region of the anterior elbow, where the nerve enters the forearm by passing between the two heads of the pronator teres muscle. The transducer was positioned transversely at the level of the humeral trochlear cartilage [15,16].

In the upper part of the ultrasound image, the brachialis muscle was distinguished along with the brachial artery, which appears as a rounded, anechoic structure. Typically, Doppler mode is not required for confirmation, as, unlike the artery, the nearby veins are easily compressed [15]. By laterally moving the probe, the brachioradialis muscle and radial nerve can be visualized. Medially, the pronator teres muscle and the MN are identified [15,16].

**Fig 2:**
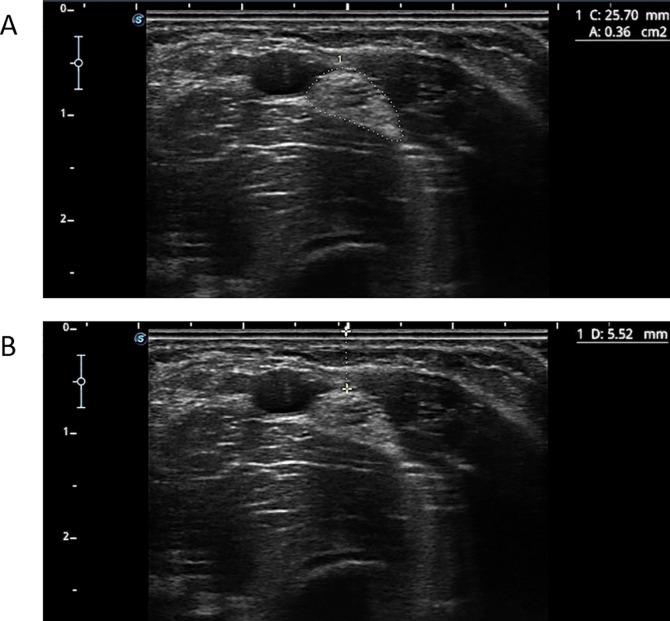
Ultrasound image of the MN where proximal measurements were performed. The cross-sectional area (CSA) (A) and the nerve-to-skin distance (B) are shown.

The transducer angle was adjusted to achieve a perpendicular position to the MN, allowing for the smallest possible cross-sectional image. To assess the CSA, the ultrasound device’s tracing tool was used to outline the inner boundary of the hyperechoic nerve edge [17] (Fig 2A). Linear measurements were also taken of the shortest distance between the MN and the skin surface (Fig 2B), as well as between the nerve and the brachial artery.

For the ultrasound evaluation of the MN at the mid-forearm, the transducer was moved distally along the axis of the limb, positioning slightly medial to the midline on the anterior surface of the forearm. At this level, the MN emerges between the humeral and ulnar heads of the pronator teres, a region referred to as the pronator tunnel. The nerve then passes through the sublimis bridge, a fibrous structure between the radial and humeral heads of the FDS muscle [15].

**Fig 3:**
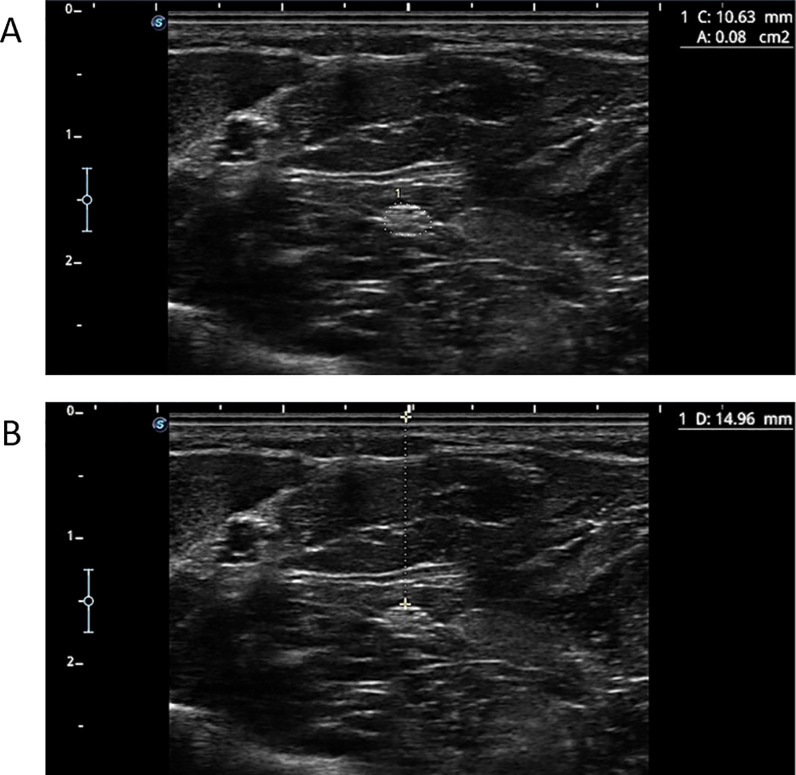
Ultrasound image of the MN where measurements were performed in the middle third of the forearm. The cross-sectional area (CSA) (A) and the nerve-to-skin distance (B) are shown.

As the MN descends along the forearm, it follows a straight path between the superficial flexor digitorum and deep flexor digitorum muscles [1,4,15]. To optimize its visualization, the transducer angle was adjusted until a clear, rounded image of the nerve was obtained, minimizing the effect of anisotropy (Fig 3A).

The CSA of the nerve was measured using the ultrasound device’s tracing function, outlining the inner hyperechoic boundary of the nerve. Additionally, calipers were used to measure the shortest distance (in mm) between the MN and the skin surface (Fig 3B) [17].

### Statistical analysis

Data analysis was conducted using SPSS v.29 for Windows (IBM, USA). Statistical significance was set at p < 0.05 for all analyses. The Kolmogorov-Smirnov test (p > 0.05) assessed data normality. Given the non-normal distribution of several variables, a logarithmic transformation (log1p) was applied before conducting the correlation analysis and hierarchical regression models to ensure statistical assumptions were met. Descriptive statistics are reported on the original (raw) scale to preserve clinical interpretability, while inferential statistics were performed on the transformed values. Post-transformation diagnostics were conducted to verify data normalization. These included visual inspection of histograms and P–P plots, confirming improved distributional characteristics and the validity of the parametric approach. Mann-Whitney U tests were applied to compare differences between sexes. Pearson correlation analysis was performed to examine the relationships between study variables. Correlation strengths were classified as negibible (<0.3), low (0.3–0.5), moderate (0.5–0.7), high (0.70–0.90) and very high (0.90 to 1) [18].

Hierarchical linear regression models were applied to identify predictors of nerve-to-skin and nerve-to-artery distances. Proximal forearm circumference was entered as the primary predictor in all models. The adjusted R^2^ change was reported after each step to evaluate the contribution of new predictors. However, models with the highest explanatory power were retained. Other anthropometric variables (age, sex, height, BMI) were initially tested but excluded from the final models due to low incremental explanatory value (ΔR^2^ < 0.01, p > 0.05) or multicollinearity (assessed using variance inflation factors (VIF), considering values below 10 as acceptable). To verify regression assumptions, residuals were examined through scatterplots of standardized residuals vs. predicted values (to assess linearity and homoscedasticity), and Q–Q plots (to assess normality). Independence of residuals was evaluated using the Durbin–Watson statistic, with values close to 2 indicating no autocorrelation.

## Results

A total of 53 participants (25 women and 28 men) were recruited for the study. The average age for the entire sample was 31.89 years, with the men averaging 31.57 years and the women averaging 32.24 years. Statistically significant differences were observed between genders in terms of weight and height (p < 0.05). Men had an average weight of 78.39 kg and an average height of 180.14 cm, while women had an average weight of 62.4 kg and an average height of 163.8 cm. The overall BMI for the sample was 23.76 kg/m^2^, with men having a BMI of 24.21 kg/m^2^ and women a BMI of 23.62 kg/m^2^ (Table 1).

**Table 1:** Anthropometric and ultrasonography features.

Variable	Total (N = 53)	Women (N = 25)	Men (N = 28)	p-value (Effect Size – Hodges-Lehmann)
**Age (years)**	31.89 ± 14.13	32.24 ± 15.22	31.57 ± 13.36	0.851 (0.25)
**Weight (kg)**	70.85 ± 12.45	62.40 ± 10.66	78.39 ± 8.52	<0.001 (6.74)
**Height (cm)**	172.43 ± 9.93	163.80 ± 5.15	180.14 ± 5.99	<0.001 (10.22)
**BMI**	23.76 ± 3.42	23.26 ± 3.91	24.21 ± 2.91	0.165 (1.12)
**Proximal forearm circumference (cm)**	26.42 ± 2.56	24.64 ± 2.31	28.00 ± 1.54	<0.001 (2.10)
**Mid-forearm circumference (cm)**	23.92 ± 2.46	22.56 ± 1.98	25.14 ± 2.22	<0.001 (2.35)
**Proximal median nerve area (cm²)**	0.1215 ± 0.0337	0.1132 ± 0.0326	0.1289 ± 0.0335	0.118 (0.008)
**Distal median nerve area (cm²)**	0.0709 ± 0.0199	0.0684 ± 0.0219	0.0732 ± 0.0181	0.123 (0.005)
**Nerve-to-skin distance (proximal) (mm)**	6.97 ± 2.81	6.40 ± 2.51	7.47 ± 3.01	0.212 (0.45)
**Nerve-to-skin distance (distal) (mm)**	16.54 ± 2.51	15.67 ± 2.31	17.32 ± 2.46	0.010 (1.25)
**Nerve-to-artery distance (mm)**	4.07 ± 3.52	3.76 ± 3.84	4.36 ± 3.25	0.354 (0.34)

### Anthropometric and ultrasonography characteristics

The descriptive data showed that men were taller and heavier than women (p < 0.001). Forearm circumference was also larger in men, both at the proximal (p < 0.001) and mid-forearm levels (p < 0.001). Regarding MN area, there were no significant differences between men and women in proximal (p = 0.118) or distal (p = 0.123) measurements. For nerve-to-skin distances, men had greater proximal and distal values than women, but only the distal measurement reached statistical significance (p = 0.010). There was no significant difference in the nerve-to-artery distance between men and women (p = 0.354) (Table 1).

### Correlation analysis

The Pearson correlation analysis revealed several significant associations (p < 0 01) among the study variables. BMI showed a strong positive correlation with proximal forearm circumference, mid-forearm circumference, and distal nerve-to-skin distance (all p < 0 001). Proximal and mid-forearm circumference were highly correlated (p < 0 001), suggesting a strong relationship between these anthropometric measures. Proximal MN area was significantly correlated with BMI, proximal forearm circumference, and mid-forearm circumference (p values ranging from 0 002–0 003). Distal nerve-to-skin distance was significantly correlated with proximal nerve-to-skin distance and nerve-to-artery distance (p < 0 001 and p = 0 003, respectively). Distal MN area did not show significant correlations with BMI, forearm perimeters, or nerve-to-skin distances (all p values > 0 05) (Table 2).

**Table 2:** Pearson correlation matrix.

	BMI	Prox. Forearm Circumf.	Mid-forearm Circumf.	Prox. Median Nerve Area	Dist. Median Nerve Area	Nerve-Skin Dist. (Prox.)	Nerve-Skin Dist. (Dist.)
**Prox. Forearm Circumf.**	0.985**	--					
**Mid-forearm Circumf.**	0.979**	0.993**	--				
**Prox. Median Nerve Area**	0.421**	0.411**	0.401**	--			
**Dist. Median Nerve Area**	−0.062	−0.091	−0.120	0.441**	--		
**Nerve-Skin Dist. (Prox.)**	0.713**	0.709**	0.707**	0.263	−0.117	--	
**Nerve-Skin Dist. (Dist.)**	0.964**	0.976**	0.975**	0.395**	−0.095	0.666**	--
**Nerve-Artery Dist.**	0.418**	0.424**	0.437**	0.110	−0.219	0.673**	0.407**

**Abbreviations:** BMI (Body Mass Index), Prox FA Circ (Proximal Forearm Circumference), Mid FA Circ (Mid-Forearm Circumference), Prox Med N Area (Proximal Median Nerve Area), Dist Med N Area (Distal Median Nerve Area), Prox N-S Dist (Proximal Nerve-to-Skin Distance), Dist N-S Dist (Distal Nerve-to-Skin Distance), N-A Dist (Nerve-to-Artery Distance). *p < 0.05; **p < 0.01.

### Hierarchical regression analysis

A hierarchical linear regression analysis was conducted to examine the association between anthropometric and nerve-related variables and three different nerve-related distances. Proximal forearm circumference significantly predicted proximal nerve-to-skin distance, explaining 49.4% of the variance (adjusted R^2^ = 0.494). The model was statistically significant (F(1,51) = 51.67, p < 0.001). For distal nerve-to-skin distance, proximal forearm circumference remained a strong predictor, accounting for 95.2% of the variance (adjusted R^2^ = 0.952; F(1,51) = 1026.99, p < 0.001). Finally, a linear regression model assessed the relationship between proximal forearm circumference and nerve-to-artery distance. While the association was statistically significant (F(1,51) = 11.18, p = 0.002), the explained variance was 16.4% (adjusted R^2^ = 0.164) (Table 3).

**Table 3:** Hierarchical regression analysis.

Step	Predictor	B	SE B	95% CI	β	t	p	R² adjusted
**Proximal nerve-to-skin distance**								
1	Prox Forearm Circumference	0.198	0.028	0.143, 0.253	0.709	7.188	<0.001	0.494
**Distal nerve-to-skin distance**								
1	Prox Forearm Circumference	0.609	0.019	0.571, 0.647	0.976	32.047	<0.001	0.952
**Artery-to-nerve distance**								
1	Prox Forearm Circumference	0.127	0.038	0.051, 0.203	0.424	3.343	0.002	0.164

### Proximal median nerve depth prediction

Using hierarchical regression analysis, a practical formula was created to determine the optimal needle length for the invasive procedure targeting the median nerve. The regression equation derived from the results is as follows:


$$\begin{array}{c} \text{\textbackslash{}emph \{Predicted depth (mm):\textbackslash{} 1.369 \{\textbackslash{}bf +}0.198\;xcircumference\;proximal\;forearm\;(cm)\;\}\} \end{array}$$

### Mid-third median nerve depth prediction

Similarly, a practical formula was developed to select the appropriate needle length for the invasive approach of the median nerve in the mid-third of the forearm. The regression equation derived from the results is as follows:


$$\begin{array}{c} \text{\textbackslash{}emph \{Predicted\textbackslash{} depth\textbackslash{} (mm):\textbackslash{} 0.885\textbackslash{} \{\textbackslash{}bf +}0.609\;x\;circumference\;proximal\;forearm\;(cm)\;\}\} \end{array}$$

## Discussion

This is the first study designed to establish reference values for the location of the MN at two points along its course in the forearm within a healthy population sample. The findings demonstrate that the proximal forearm circumference is a strong and reliable predictor of the depth of the MN relative to the skin, particularly at the forearm level. The predictive model showed exceptional accuracy, explaining more than 95% of the variance in the distal nerve-skin distance. In contrast, although the relationship between forearm circumference and nerve-artery distance was statistically significant, its explanatory power was modest, suggesting that additional anatomical or physiological factors may influence this relationship.

Previous research has examined the relationship between the CSA of the MN at various locations and demographic factors, yielding contradictory results. While some studies have identified significant correlations between the CSA of the MN at the distal wrist fold and variables such as age, height, weight, and BMI [19,20], others have found no such associations [21–23]. Similarly, studies examining this variable at the mid-forearm level did not find significant correlations with height or BMI [24,17]. These discrepancies may be due to variations in the definition of the evaluated anatomical points, as well as differences in the characteristics of the studied populations.

Furthermore, it has been reported that performing hand activity may influence the variability of the CSA of the MN. A previous study [25] observed an increase in the nerve’s transverse diameter at the wrist level following standardized cutting activity, with a gradual reduction in 5–10 minutes thereafter. This suggests that the timing of the assessment and previous activities may affect the results.

The findings of this study are particularly relevant for preventing accidental punctures during invasive procedures targeting the MN. Quantifiable and ecological factors, such as BMI and forearm circumference, were significantly correlated with the nerve’s depth. In particular, BMI was identified as a determining factor, with higher values,suggesting larger subcutaneous adipose tissue and deeper neurovascular structures [26].

In the field of rehabilitation, invasive techniques are common for treating musculoskeletal and nervous system disorders [27]. However, complications such as radial and MN neuropathies induced by puncture have been documented on several occasions [28,29]. While ultrasound may reduce the risk of nerve injury, its application is not widespread due to limitations in training and access to high-resolution technology [30,31]. Therefore, predictive models that estimate the nerve’s location are of great clinical interest.

The predictive model developed in this study provides specific recommendations for selecting needle length based on forearm circumference. For proximal MN access, a 13 mm needle is recommended for patients with a proximal third forearm circumference ≤ 26 cm, and a 25 mm needle for those with a larger circumference. At the mid-forearm level, a 25 mm needle is suggested for patients with a circumference < 24 cm, and a 30 mm needle for circumferences ≥ 24 cm.

By applying the derived formulas, healthcare professionals can ensure that needles reach the appropriate depth, improving the effectiveness of treatments and minimizing the risk of complications. These tools may be especially useful in managing conditions affecting the MN, such as carpal tunnel syndrome, a condition affecting 7–19% of the population [32]. Accurately determining the nerve’s depth could optimize interventions, improving clinical outcomes.

These findings have important clinical implications, highlighting the potential value of incorporating simple anthropometric measurements to enhance the precision and safety of ultrasound-guided procedures involving the MN. Such predictive models could reduce procedure risks and improve therapeutic outcomes by enabling clinicians to anticipate anatomical variations based on external body metrics.

Although the predictive model developed in this study represents a promising tool, its findings are based on a healthy cohort, and further testing is necessary for external validation. Future research should focus on validating these results in broader populations, including individuals with different age ranges, body compositions, and pathological conditions. Additionally, integrating complementary variables, such as muscle mass, subcutaneous tissue thickness, or biomechanical properties, could further refine the predictive capacity of the models presented.

### Limitations

The study’s limitations include a homogeneous sample and unexamined variability. All participants were healthy, white-skinned adults aged 18–60, which limits generalizability to other skin color groups, pediatric populations, or individuals with median nerve pathologies. Additionally, the model did not account for certain anatomical and ultrasound-related variability – for example, differences in tissue echogenicity, body composition, limb biomechanics, or physical activity level – and no detailed physical exams were conducted so that subclinical neuropathies might have gone undetected; these unconsidered factors could have affected the model’s accuracy. The research also lacked interobserver reliability assessment: a single examiner performed all ultrasound measurements, preventing verification of measurement reproducibility and limiting the model’s external validity. Finally, the model showed limited predictive power for one critical measure (explaining only about 16% of the variance in nerve–artery distance), implying that additional anatomical or physiological factors must be integrated to improve accuracy and ensure safer, risk-free interventions.

## Conclusions

This study demonstrates that forearm circumference is a key factor in predicting the depth of the MN at two specific points along its path. The developed regression models could accurately estimate the distance between the nerve and the skin, which may enhance the safety of invasive procedures. These findings highlight the clinical value of using easily obtainable body measurements to support nerve localization, especially in settings where ultrasound is unavailable or impractical.

The models may help guide decision-making in pain management, and they offer a simple, low-cost tool for improving the precision of interventions. Furthermore, these models can be incorporated into educational settings to support the training of clinicians and the development of safer procedural protocols.

## Supporting information

S1 File Title page. (DOCX)

S2 File Informed consent. (DOCX)
