# Correction: Predicting median nerve depth from anthropometric features: A tool for safer invasive procedures

**DOI:** 10.1371/journal.pone.0338241

**Published:** 2025-12-05

**Authors:** Sara Mogedano-Cruz, Ángel González-de-la-Flor, Cristina Rodríguez-Anadón, Lucimere Bohn, Jorge Villafañe, Carlos Romero-Morales

The Funding statement for this article is incorrect. The correct Funding statement is as follows: This project was funded through a competitive call, specifically the 5th Call for Research Grants awarded by Ilustre Colegio Profesional de Fisioterapeutas de la Comunidad de Madrid.

## References

[1] Mogedano-Cruz S, González-de-la-Flor Á, Rodríguez-Anadón C, Bohn L, Villafañe J, Romero-Morales C. Predicting median nerve depth from anthropometric features: A tool for safer invasive procedures. PLoS One. 2025;20(8):e0330383. doi: 10.1371/journal.pone.0330383 40824904 PMC12360588

