# Supplementary material for: Predicting median nerve depth from anthropometric features: A tool for safer invasive procedures
Source: PLoS One. 2025 Aug 18;20(8):e0330383. doi: 10.1371/journal.pone.0330383 (PMC12360588; doi:10.1371/journal.pone.0330383)
Supplement: S1 File — (DOCX) [file pone.0330383.s001.docx]

**Predicting median nerve depth from anthropometric features: a tool for safer invasive procedures**

**AUTHORS**

Sara Mogedano-Cruz (1), [sara.mogedano@universidadeuropea.es](mailto:sara.mogedano@universidadeuropea.es)

Ángel González-de-la-Flor (1) [angel.gonzalez@universidadeuropea.es](mailto:angel.gonzalez@universidadeuropea.es)

Cristina Rodríguez-Anadón (1) [cristina.rodriguezanadon@gmail.com](mailto:cristina.rodriguezanadon@gmail.com)

Lucimere Bohn (2) [lucimerebohn@gmail.com](mailto:lucimerebohn@gmail.com)

Jorge Villafañe (1) [mail@villafane.it](mailto:mail@villafane.it)

Carlos Romero-Morales (1), [carlos.romero@universidadeuropea.es](mailto:carlos.romero@universidadeuropea.es)

**INSTITUTIONS**

1. Department of Physiotherapy. Faculty of Medicine, Health and Sports. European University of Madrid. Villaviciosa de Odón, 28670, Madrid, Spain.
2. Lusófona University, Rua Augusto Rosa, 24, Porto, Portugal

**ADRESS FOR REPRINT REQUESTS / CORRESPONDING AUTHOR**
