# Supplementary material for: Predicting median nerve depth from anthropometric features: A tool for safer invasive procedures
Source: PLoS One. 2025 Aug 18;20(8):e0330383. doi: 10.1371/journal.pone.0330383 (PMC12360588; doi:10.1371/journal.pone.0330383)
Supplement: S2 File — (DOCX) [file pone.0330383.s002.docx]

**INFORMATION SHEET**

**Title of the study:** Predicting Median Nerve Depth from Anthropometric Features: A Tool for Safer Invasive Procedures

**Promoter:** Sara Mogedano

**Center:** European University of Madrid

We are writing to inform you about a research study that will be conducted at the European University of Madrid, in which you are invited to participate. The purpose of this document is to provide you with the correct and necessary information to evaluate whether or not you wish to participate in the study. Below, we will explain in detail all the objectives, benefits, and potential risks of the study. If you have any questions after reading the following clarifications, we will be available to answer any questions you may have. Finally, you may discuss your participation with anyone you deem appropriate.

**What is the objective of this study?**

This study aims to quantitatively assess the location of the median nerve and adjacent structures at the forearm to predict the correct application and safety of invasive procedures.

**Study summary**

If you decide to participate in the study, the following steps will be followed:

1. Explanation of informed consent, resolution of possible doubts and signing of informed consent.

2. All participants will undergo an evaluation consisting of metric and ultrasound measurements of the forearm area.

**VOLUNTARY PARTICIPATION AND WITHDRAWAL FROM THE STUDY:** Participation in this study is voluntary, so you may decide not to participate. If you decide to participate, you may withdraw your consent at any time. If you decide to withdraw from the study, you may do so by allowing the data obtained up to that point to be used for the purpose of the study, or, if you so choose, all records and data will be deleted from the computer files.

**Who can participate?**

Healthy subjects between 18 and 60 years of age.

**What are the potential benefits and risks of my participation?**

At the end of the research, you may be informed, if you wish, of the main results and general conclusions of the study.

There are no risks or benefits to the patient's health.

**Who has access to my personal data and how is it protected?**

The processing, communication, and transfer of personal data of all participating subjects will comply with the provisions of Organic Law 3/2018 of December 5, on the Protection of Personal Data and the Guarantee of Digital Rights. In accordance with the aforementioned legislation, you may exercise your rights of access, modification, objection, and deletion of data, for which you should contact your study physician.

The data collected for the study will be identified by a code, and only the research team responsible for the study will be able to associate this data with you. Therefore, your identity will not be revealed to anyone except in exceptional circumstances, in the case of a legal requirement. Only the data collected for the study will be transmitted to third parties and other countries. Under no circumstances will this information contain information that could directly identify you, such as your first and last name, initials, address, social security number, etc. If this transfer occurs, it will be for the same purposes as the study described, guaranteeing confidentiality at least at the level of protection provided by current legislation in our country.

**Will I receive any financial compensation?**

No

**Who is funding this research?**

There is no funding

**OTHER RELEVANT INFORMATION**

If you decide to withdraw your consent to participate in this study, no new data will be added to the database, and you may request the destruction of your data and/or all previously retained identifiable records to prevent further analysis. You should also be aware that you may be excluded from the study if the study investigators deem it appropriate, whether for safety reasons, due to an adverse event, or because they believe you are not complying with established procedures. In either case, you will receive an adequate explanation of the reason for your withdrawal from the study.

**SCIENTIFIC QUALITY AND ETHICAL REQUIREMENTS OF THE STUDY**

This study has been registered with the Research Commission of the European University of Madrid, Valencia and the Canary Islands, which oversees the scientific quality of research projects conducted at the center. When research is conducted with human subjects, this Commission ensures compliance with the provisions of the Declaration of Helsinki and current legal regulations on biomedical research (Law 14/2007, of June, on biomedical research) and clinical trials (Royal Decree 1090/2015, of December 4, regulating clinical trials with medicinal products).

**QUESTIONS**

At this point, we give you the opportunity, if you haven't done so before, to ask any questions you deem appropriate. The research team will answer them to the best of their ability.

**STUDY RESEARCHERS**

If you have any questions about any aspect of the study or would like to comment on any of this information, please do not hesitate to ask the research team members. If, after reading this information and clarifying any questions, you decide to participate in the study, you must sign your informed consent form.

**INFORMED CONSENT:**

Mr./Ms. ____________________________________________________, aged _____, with National Identity Document (DNI) and address at ____________________________________, hereby certify that I have received a satisfactory explanation of the study procedure, its purpose, risks and benefits.

I am satisfied with the information I received, I understand it, all my questions have been answered, and I understand that my participation is voluntary.

I give my consent to the proposed procedure and I am aware of my right to withdraw it at any time, with the sole obligation of informing the researcher responsible for the study of my decision.

In Madrid, on the day______ of _______________ of _______.

__________________________________ _________________________________

Researcher's signature Signature and ID number of the participant
